# Effect of surface polishing on roughness, biofilm formation, and biocompatibility of LCD-printed denture base polymer

**DOI:** 10.1038/s41598-026-45942-y

**Published:** 2026-03-31

**Authors:** Amanda Costa Ferro, Jonatas Silva de Oliveira, Lais Scabelo, Leonardo Viana Araújo, Carlos Mota, Matthew B Baker, Janaina Habib Jorge

**Affiliations:** 1 Department of Dental Materials and Prosthodontics, School of Dentistry, São Paulo State University (UNESP), Humaita, 1680, Araraquara, 14801-903 Brazil; 2 Department of Complex Tissue Regeneration, Institute for Technology-Inspired Regenerative Medicine (MERLN), Maastricht University, Maastricht, The Netherlands; 3 School of Dentistry, Ceuma University (UNICEUMA), São Luís, Maranhão Brazil; 4 Department of Instructive Biomaterials Engineering, Institute for Technology-Inspired Regenerative Medicine (MERLN), Maastricht University, Maastricht, The Netherlands

**Keywords:** Polymers, *Candida albicans*, Surface properties, Printing, Three-dimensional, Biotechnology, Health care, Materials science, Medical research

## Abstract

This study investigated the effect of surface polishing on roughness, mechanical performance, and biological properties of a liquid crystal display (LCD)‑printed denture base polymer. Disc‑shaped samples of three-dimensional (3D) printing polymer were fabricated and allocated to polished (P) or not polished (NP) groups for comparative analysis. Polishing was performed manually in a standardized manner using sequential silicon carbide sandpapers, while the NP samples were sanded to reproduce the texture generated by the printing lines. Surface roughness was measured by contact and non‑contact profilometry, and topography analyzed by scanning electron microscopy (SEM). Mechanical properties were assessed by compressive stress testing and wettability by water contact angle. Biological assays included *C. albicans* biofilm proliferation (colony-forming units, CFU), metabolism (Alamar blue assay) and coverage (Live/Dead). Cytotoxicity tests with L‑929 fibroblasts under direct and indirect contact were also performed. Protein adsorption was evaluated using Alexa Fluor 488‑labeled Bovine Serum Albumin (BSA). Data were analyzed with Student’s t‑test (α = 0.05). Polishing significantly reduced roughness to the clinically acceptable threshold, with SEM and laser microscopy confirming reduction of irregularities. Mechanical testing showed similar Young’s modulus values between groups, while polished samples exhibited higher contact angles. Polished surfaces reduced *C. albicans* biofilm metabolism and coverage on the samples, although CFU counts did not differ significantly. Cytotoxicity tests demonstrated cell metabolism comparable to the control group (100% viable cells) over time. Protein adsorption was equivalent between groups. In conclusion, polishing reduced surface roughness and *C. albicans* metabolism and coverage on LCD‑printed denture base, while maintaining mechanical properties and cytocompatibility.

**Graphical abstract:**

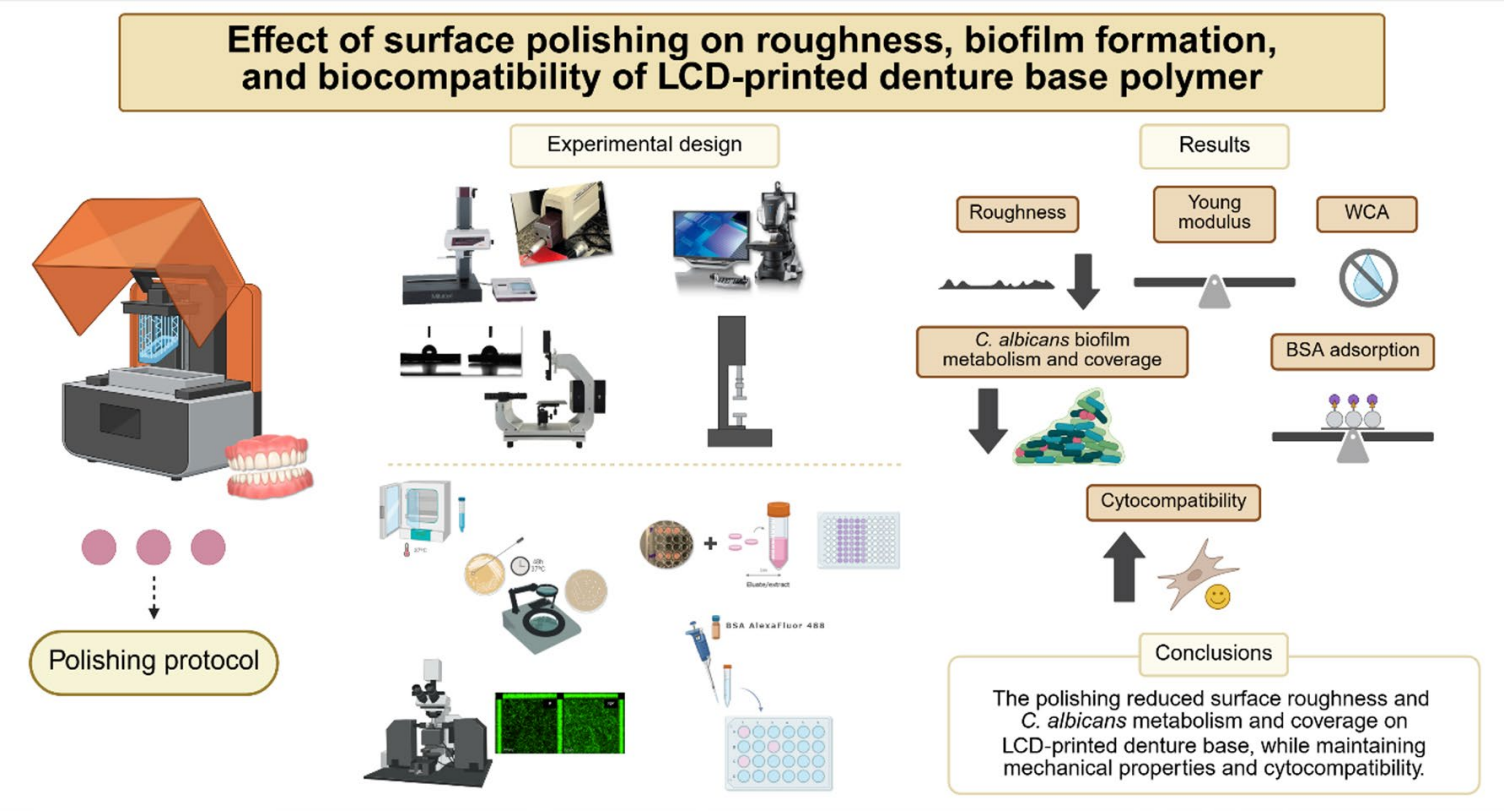

**Supplementary Information:**

The online version contains supplementary material available at 10.1038/s41598-026-45942-y.

## Introduction

Denture stomatitis (DS) is an inflammatory condition affecting up to 65% of denture wearers^1^. It is more frequent among hospitalized elderly individuals, smokers, and patients with systemic conditions^2–4^. Although its etiology is multifactorial, *Candida albicans* plays a central role by adhering to denture surfaces, forming biofilms, and expressing virulence factors that facilitate tissue invasion^5^. Because the denture base acts as a reservoir for *Candida*, its surface characteristics are decisive in either preventing or aggravating DS^1^.

Prevention of DS have focused primarily on the denture, since *Candida* spp. are more frequently isolated from the internal surface of dentures than from the corresponding mucosa^1,6^. In clinical practice, however, the intaglio surface of conventional dentures is rarely polished due to concerns about reduced retention. In fact, this is mainly determined by the border and posterior palatal seals, which stabilize the saliva film and create a suction effect. Surface tension at the periphery also contributes, but the key factors are accurate base adaptation and an effective border seal^7^.

Consistently, a recent study by Neeraja et al.^8^ evaluated the influence of surface roughness on the retention of complete dentures fabricated with different materials and techniques. The authors reported an inverse correlation between roughness and retention, with smoother denture base surfaces yielding higher retention values. This observation has led to proposals for polishing the inner denture surface as a strategy to minimize biofilm accumulation.

The correlation between surface roughness and *Candida* adhesion to heat-polymerized polymethyl methacrylate (PMMA) resins remains controversial^9,10^. While Izumida et al.^11^ found no significant difference in biofilm formation between surfaces of different roughness, other studies observed higher fungal adhesion on rough substrates^12,13^. Yamauchi et al.^14^ also showed that topographical irregularities increased *C. albicans* adhesion and proliferation, reinforcing the need for smooth intaglio surfaces to reduce microbial colonization on denture bases^6,15,16^.

Beyond roughness, other physicochemical properties of denture base materials can influence microbial adhesion and biofilm formation^17^. Surface charge may modulate binding forces between microorganisms and the substrate, while also affecting protein adsorption and salivary pellicle development. Wettability and surface free energy are also relevant. For example, microorganisms often show affinity for hydrophobic surfaces. Moreover, roughness can amplify wetting properties, enhancing the intrinsic character of the material^18,19^.

3D printing has become an effective method for producing denture bases, reducing material waste and fabrication costs compared with conventional techniques^20–22^. Among these technologies, liquid crystal display (LCD) printing is widely used in clinical practice because it is considered a faster and lower-cost system^23^. However, due to the layer-by-layer printing process and lower accuracy than digital light processing (DLP) and stereolithography (SLA), a more pronounced stair-step phenomenon has been reported, contributing to increased roughness of 3D-printed objects^24^.

These irregularities may promote microbial accumulation if not properly finished, demonstrating the importance of establishing finishing protocols that smooth the surface to clinically acceptable levels. Quirynen et al.^25^ reported that roughnessabove Ra = 0.2 μm significantly increase microbial adhesion, while reductions below this threshold do not provide additional benefit. This limit has since been used as a reference for polishing procedures on 3D-printed polymers, although studies mainly addressed surface roughness without evaluating microbiological outcomes^26^.

Despite the widespread adoption of 3D printing in prosthodontics, the existing literature has not fully addressed the fact that the printing process itself generates surface texture, even though digital impressions aim to capture oral anatomy accurately. In addition, finishing and polishing procedures for additively manufactured denture bases are not consistently standardized for clinical application, leaving uncertainty about how best to achieve clinically acceptable surface quality.

To date, no studies have evaluated the effect of polishing protocols on LCD‑printed denture bases with respect to *C. albicans* adhesion and related properties. Therefore, this study aimed to investigate whether surface polishing influenced surface roughness as well as the mechanical and biological performance of an LCD‑printed denture base polymer. The null hypothesis was that polishing would not significantly affect surface roughness and topography, compressive stress, wettability, *C. albicans* adhesion and biofilm formation or cytocompatibility.

## Materials and methods

### Preparation of samples

The sample size for each test was defined according to previously published in vitro studies with similar experimental design^27–30^. Circular samples (14 mm x 1.2 mm) were virtually designed and sliced (Autodesk Meshmixer v.3.5.474, Autodesk, Inc; Anycubic Photon Workshop V2.1.24), followed by the printing (Anycubic Photon Mono SE printer, Shenzhen Anycubic Technology Co., Ltd.) with a commercially available polymer for denture base (Cosmos Denture, Yller Biomaterials SA). The samples were printed at a 90‑degree print orientation with a layer thickness of 0.05 mm, based on Shim et al.^31^. After printing, the cleaning process was performed with isopropyl alcohol under agitation (Form Wash, Formlabs), followed by post‑curing in a UV light-curing unit (Form Cure, Formlabs), strictly following the manufacturer’s instructions (see Supplementary Information for details).

### Polishing procedure

After preparation, samples were divided into polished (P) and not polished (NP) groups. For the NP group, a preliminary evaluation determined the average roughness of LCD‑printed samples. This value was reproduced in a standardized manner using 80‑grit silicon carbide sandpaper, ensuring all NP samples presented comparable roughness representative of the inherent texture generated by the printing lines, allowing consistent comparison with the polished group. Polishing of the P group was performed using a sequential silicon carbide sandpaper procedure (200-, 400-, 600-, 800-, 1000-, and 1200-grit), adapted from de Foggi et al.^9^, with the aim of achieving the clinically acceptable threshold for surface roughness (0.2 μm)^25^.

All polishing steps were carried out manually under continuous running water to minimize heat generation and debris accumulation. Each sanding stage was timed with a digital timer (30–60 s) and executed with uniform circular movements under light and constant pressure, controlled through previous operator training. A single trained operator conducted all procedures to reduce variability and to ensure reproducibility across all samples. Sandpaper sheets were replaced regularly to maintain abrasive efficiency. After polishing, samples were cleaned in an ultrasonic bath for 10 min and stored in distilled water at 37 °C for 48 h before testing.

### Surface roughness (Ra)

Subsequently, the samples were evaluated by both contact (SJ 400, Mitutoyo Corp.) and non-contact (VK-X250 3D Laser Scanning Microscope, Keyence) profilometers. Contact measurements (*n* = 9) were conducted using 5 μm radius diamond tip at speed of 0.5 mm/s, with resolution of 0.01 μm, 0.8 mm interval, and 2.4 mm transverse length. Three readings were performed per sample across predetermined, representative areas. For non-contact analysis (*n* = 2), Differential Interference Contrast (DIC) optical images were acquired at 10× and 20× magnifications. Then, Ra was quantified using the MultiFile Analyzer software based on three representative images of distinct locations per sample to determine mean values.

### Surface topography

The surface topography was evaluated by Scanning Electronic Microscopy (SEM) analysis. Samples from each group (*n* = 1) were dehydrated, metallized with carbon, and positioned in the microscope (JEOL JSM, 6610LV) to obtain the images at final magnifications of 100×, 250×, and 500×.

### Water contact angle (WCA)

The wettability of the samples was evaluated using a drop-shape analyzer (Krüss DSA25 system). A single droplet of deionized water (0.40 µL) was deposited on each sample (*n* = 9). After 10 s of contact, images were captured and analyzed. The WCA was calculated with the DSA4 software^32^.

### Compressive stress

The compressive test was performed using an ElectroForce 3200 mechanical tester (TA Instruments) equipped with a 450 N load cell and operated at a strain rate of 1%, simulating localized compressive loads on denture bases during mastication. Circular samples (P group: *n* = 6; NP group: *n* = 7) were prepared with 3 mm diameter and 1 mm thickness. The linear elastic region was defined as the portion of the stress–strain curve showing proportionality between these two parameters, confirmed by linear regression (R² ≥ 0.99). After testing, the interval between 15% and 18% strain met this criterion (R² = 0.99), and the slope of this region was subsequently used to calculate the Young’s modulus (MPa)^33,34^. When evident inconsistencies were observed in stress–strain curves, the data were excluded.

### Adhesion and biofilm formation of *C. albicans*

This test consisted of cultivating *Candida albicans* (ATCC 90028) biofilms on the polymer samples, subsequently assessed by cell proliferation (colony-forming units, CFU/mL) and metabolism (AlamarBlue assay) (*n* = 9, each test)^35^.

*Candida albicans* was initially cultured on Sabouraud Dextrose Agar (SDA) for 48 h at 37 °C. A standardized inoculum was prepared by incubating five colonies in 10 mL of Yeast Nitrogen Base (YNB) for 16 h at 37 °C, followed by a 1:20 dilution in YNB for an additional 8 h. The resulting suspension equivalent to 1 × 10^6^ CFU/mL was centrifuged, washed in Phosphate-Buffered Saline (PBS), and resuspended in RPMI-1640 medium. The samples were UV-disinfected (20 min/side) and placed in 24-well plates for a 90-min adhesion phase with 750 µL of the fungal suspension^36^. Non-adherent cells were removed by PBS washing, followed by a 48-h biofilm maturation period in 1500 µL of RPMI. Mature biofilms were harvested by timing the scraping to 1 min under standardized conditions, ensuring consistent detachment across all samples. Subsequently, serial dilutions (10^− 1^ to 10^− 4^) were plated onto SDA and incubated for 48 h at 37 °C to determine viable cell counts (CFU/mL).

*C. albicans* cell metabolism was analyzed using the alamarBlue^®^ reagent (Invitrogen™). After 48 h of mature biofilm formation on the samples, 10% (v/v) of alamarBlue^®^ solution was added to the plate and incubated for 4 hours^37^. Subsequently, fluorescence intensity was measured using Fluoroskan Ascent plate reader (Thermo Fisher Scientific) at 560 nm and 590 nm.

### Live-stained *C. albicans* biofilm coverage and CLSM analysis

*C. albicans* biofilm coverage was assessed using the LIVE/DEAD™ FungaLight™ Yeast kit (Invitrogen™). A working solution was prepared by mixing SYTO-9 and propidium iodide (PI) at a 1:1 ratio, subsequently diluted 1:1000 in PBS. Samples (*n* = 3) were incubated with the staining solution for 30 min at 37 °C in the dark. Images were acquired using a confocal laser scanning microscope (CLSM 800 with Airyscan, Zeiss) at 10× magnification.

Fluorescence was detected using excitation/emission wavelengths of 488/500–550 nm for SYTO-9 and 561/580–650 nm for PI. Quantification was performed using the ZEN software by defining a single region of interest (ROI), which was consistently applied to three representative images per sample. Even though both channels were monitored, the green channel (SYTO-9) was selected for quantification and figure representation to assess viable biofilm coverage. Data were expressed as mean fluorescence intensity (arbitrary units, a.u.). The red channel (PI) was utilized exclusively to confirm cell viability across the samples.

### Cytotoxicity tests

Cytotoxicity was evaluated via cell metabolism through indirect and direct exposure tests (*n* = 3, each test) following ISO 10993-5:2009 and ISO 10993-12:2021 standards^38–40^. L-929 mouse fibroblasts (NCTC clone 929 [L cell, L-929, derivative of strain L], CCL-1) were maintained in Dulbecco’s Modified Eagle medium (DMEM, Thermo Fisher Scientific) supplemented with 10% fetal bovine serum (FBS) and 1% penicillin-streptomycin. For all assays, cells (passage 13) were seeded at 5 × 10^3^ cells/well in 96-well plates for 24 h at 37 °C.

For the indirect assay, extracts were prepared by incubating the UV-disinfected samples in 3 mL of DMEM for 24 h at 37 °C. The control group consisted of 3 mL of medium stored under the same conditions. Cells were exposed to the extracts or medium for 24 h, with daily reapplication over 1, 3, and 7 days (exhaustive method). In the direct assay, the discs were punched (6 mm diameter x 1.2 mm thickness) and placed in 96-well plates, and cells were seeded directly onto the samples following the same cell density as previously described, with medium renewal every 48 h.

Metabolic activity was quantified at days 1, 3, and 7 by adding 10% (v/v) of PrestoBlue^®^ and subsequent 90 min of incubation. Fluorescence was measured (Excitation/Emission: 560/590 nm) using CLARIOstar plate reader (BMG LABTECH). Results were normalized to the control group (100% viable cells) and interpreted according to ISO thresholds^39,40^. Cell morphology and proliferation were qualitatively monitored via inverted microscopy (Nikon Eclipse Ti-S/L100, Nikon Instruments) and representative images were acquired at a selected time point.

### Qualitative and quantitative BSA adsorption

BSA adsorption was evaluated by incubating samples in 1 mL of PBS for 24 h at 37 °C, followed by an incubation in 1 mL of Alexa Fluor 488-labeled Bovine Serum Albumin (BSA) (0.01 mg/mL in PBS) for 24 h at 37 °C. Unbound protein was removed through five consecutive PBS rinses (1 mL each). Samples (*n* = 3) were imaged via fluorescence microscopy at 20× magnification^32^. Protein adsorption on the surface was quantified from three representative images using ImageJ software (NIH) by calculating the fluorescence intensity (arbitrary units, a.u.) within defined ROI.

### Statistical analysis

Data were subjected to descriptive analysis and assessed for normality and homogeneity of variances using the Shapiro-Wilk and Levene’s tests, respectively. Outliers, if any, were identified and excluded using the Grubbs’ test (extreme studentized deviate method) implemented in the GraphPad Prism outlier calculator. Despite the relatively small sample size, the data presented normal distribution and equal variance, supporting the use of a parametric test. Accordingly, the Student’s t‑test was applied for group comparisons at a significance level of 5% (α = 0.05). The number of statistical comparisons was limited to those directly addressing the study objectives, thereby minimizing the risk of type I error inflation. All analyses were performed in GraphPad Prism version 10.3.1 for Windows (GraphPad Software).

## Results

**Fig. 1:**
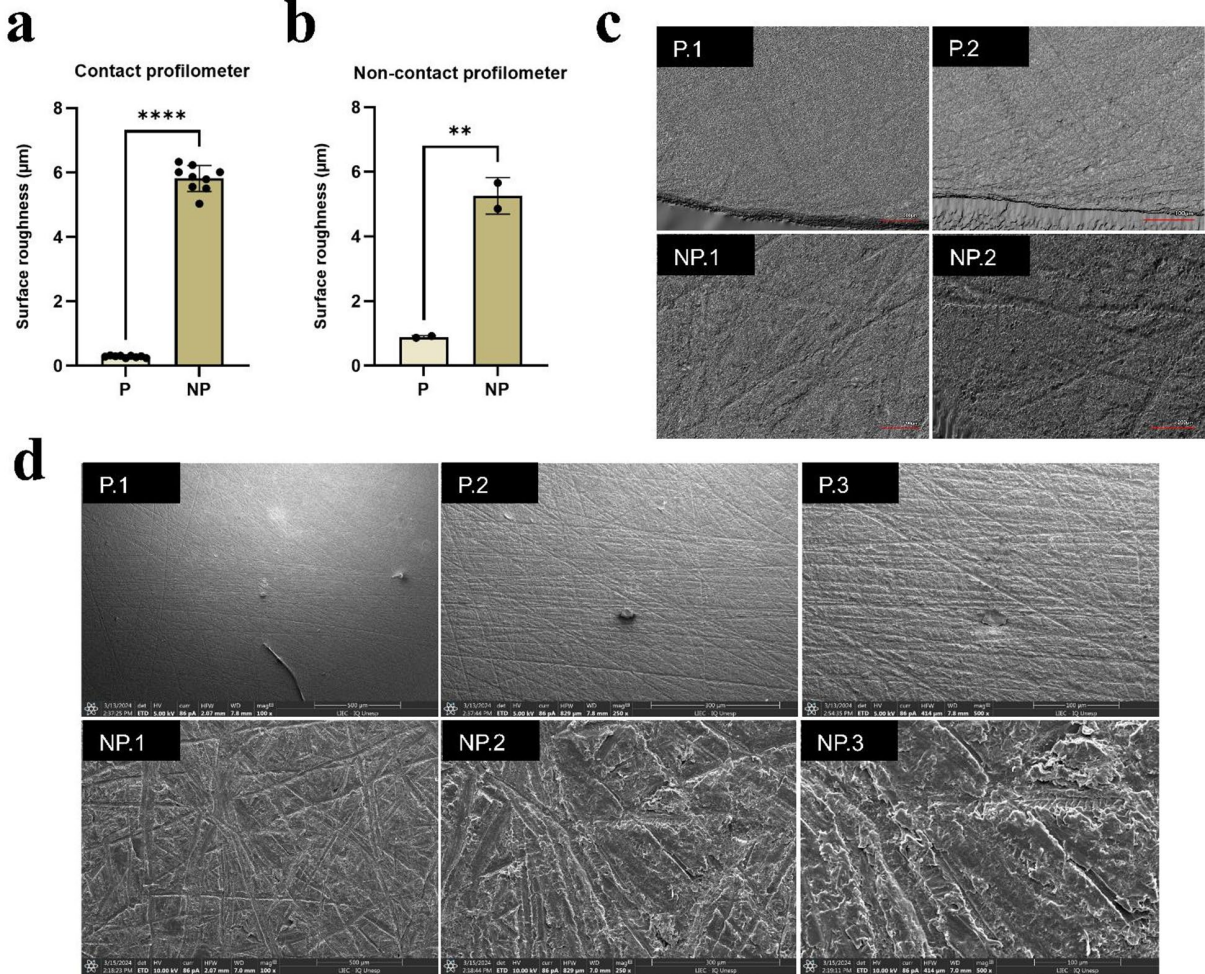
**a** Surface roughness (Ra) values measured using a contact profilometer and **b** a non-contact profilometer. **c** Representative optical microscopy images of surface topography. **d** Representative SEM micrographs of the samples. *Note.* P: polished group; NP: not polished group. Optical microscopy images were obtained at 10 × (1) and 20 × (2) magnifications (scale bars: 200 μm and 100 μm). SEM images were acquired at 100 × (1), 250 × (2), and 500 × (3) magnifications (scale bars: 500 μm, 300 μm, and 100 μm). ****p < 0.001; **p = 0.008.

The surface roughness of the polished (P) and not polished (NP) groups was evaluated using both contact and non-contact profilometers, and the data sets were analyzed independently. The Ra values obtained were 0.28 ± 0.03 μm and 5.81 ± 0.40 μm (Fig. 1a), and 0.86 ± 0.03 μm and 5.25 ± 0.56 μm (Fig. 1b), respectively. A statistically significant difference was observed between the groups, with the polishing protocol reducing Ra by approximately twenty-fold (*p* < 0.05). This distinction was confirmed qualitatively through optical microscopy and SEM images, which demonstrated pronounced irregularities in the NP group compared to the smoother and more homogeneous topography of the P samples (Fig. 1c and d).

**Fig. 2:**
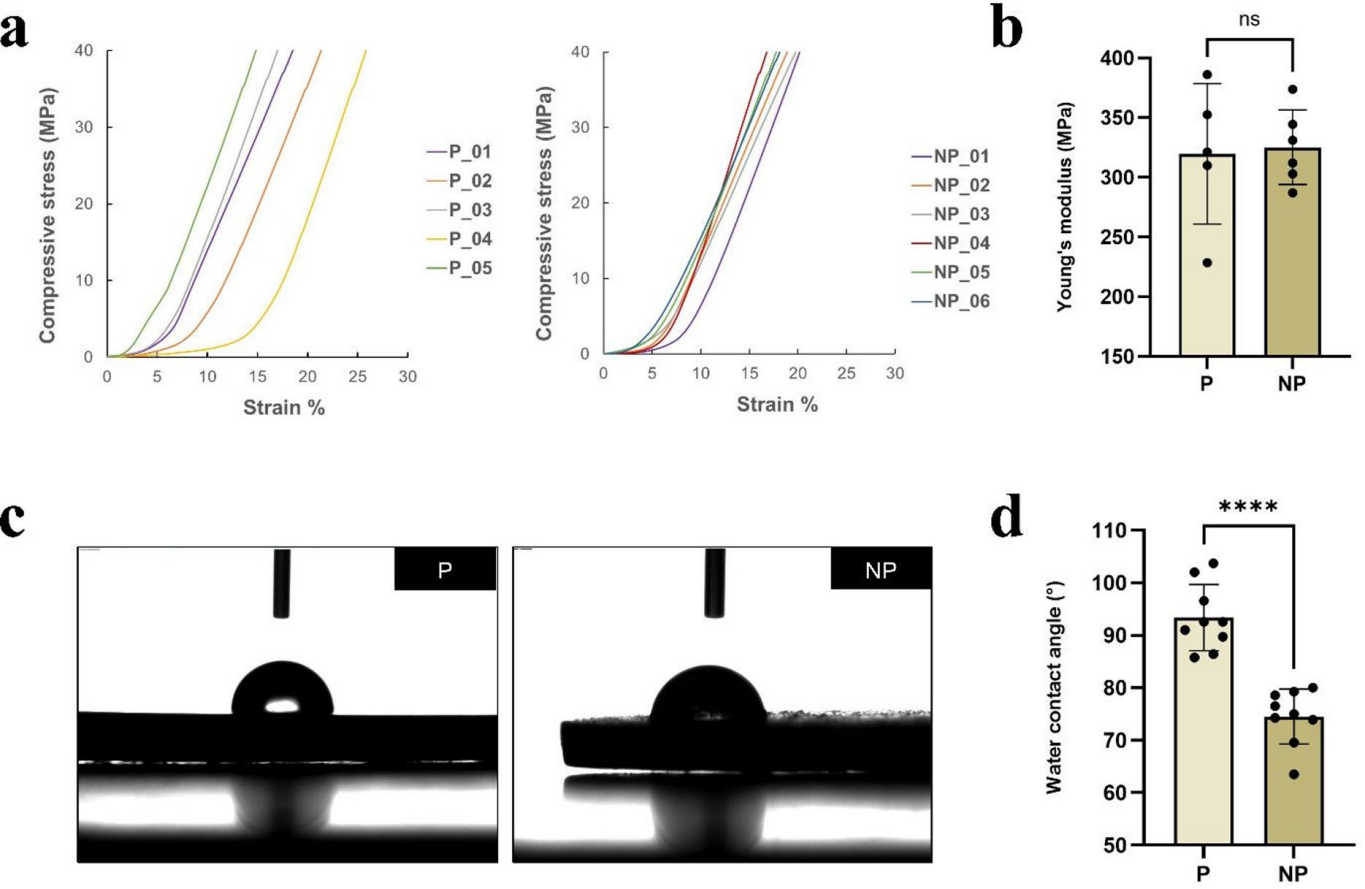
**a** Representative compressive stress–strain curves of polished (P) and not polished (NP) samples. **b** Young’s modulus values (MPa) calculated from the linear region between 15% and 18% strain (R^2^ = 0.99). **c** Representative droplet profiles used for water contact angle (WCA) measurement. **d** WCA values (θ = °) for both groups. *Note.* P: polished group; NP: not polished group. ****p < 0.001; ns: non-significant.

The stress–strain curves of the P group exhibited variable deformation, while NP group showed more consistent behavior within the group. The mean percentage strain at maximum stress was similar between the groups: 14.96% for P and 14.40% for NP (Fig. 2a). One sample from each group displayed anomalous stress–strain curves compared to the other samples and were excluded from the analysis, without formal outlier testing, as detailed in the Supplementary Information. For the calculation of Young’s modulus, a linear region of the stress–strain curve between 15% and 18% strain was selected, as this interval provided the most stable slope with R^2^ = 0.99. From this region, Young’s modulus was calculated as 319.80 ± 58.92 MPa for the P group and 325.30 ± 31.22 MPa for the NP group, with no statistically significant difference (*p* = 0.845) (Fig. 2b). For WCA analysis, the droplet profile is shown in Fig. 2c. Polished samples exhibited significantly higher water contact angle values (θ = 93.37° ± 6.30°) compared with NP samples (θ = 74.52° ± 5.24°) (*p* < 0.05) (Fig. 2d).

**Fig. 3:**
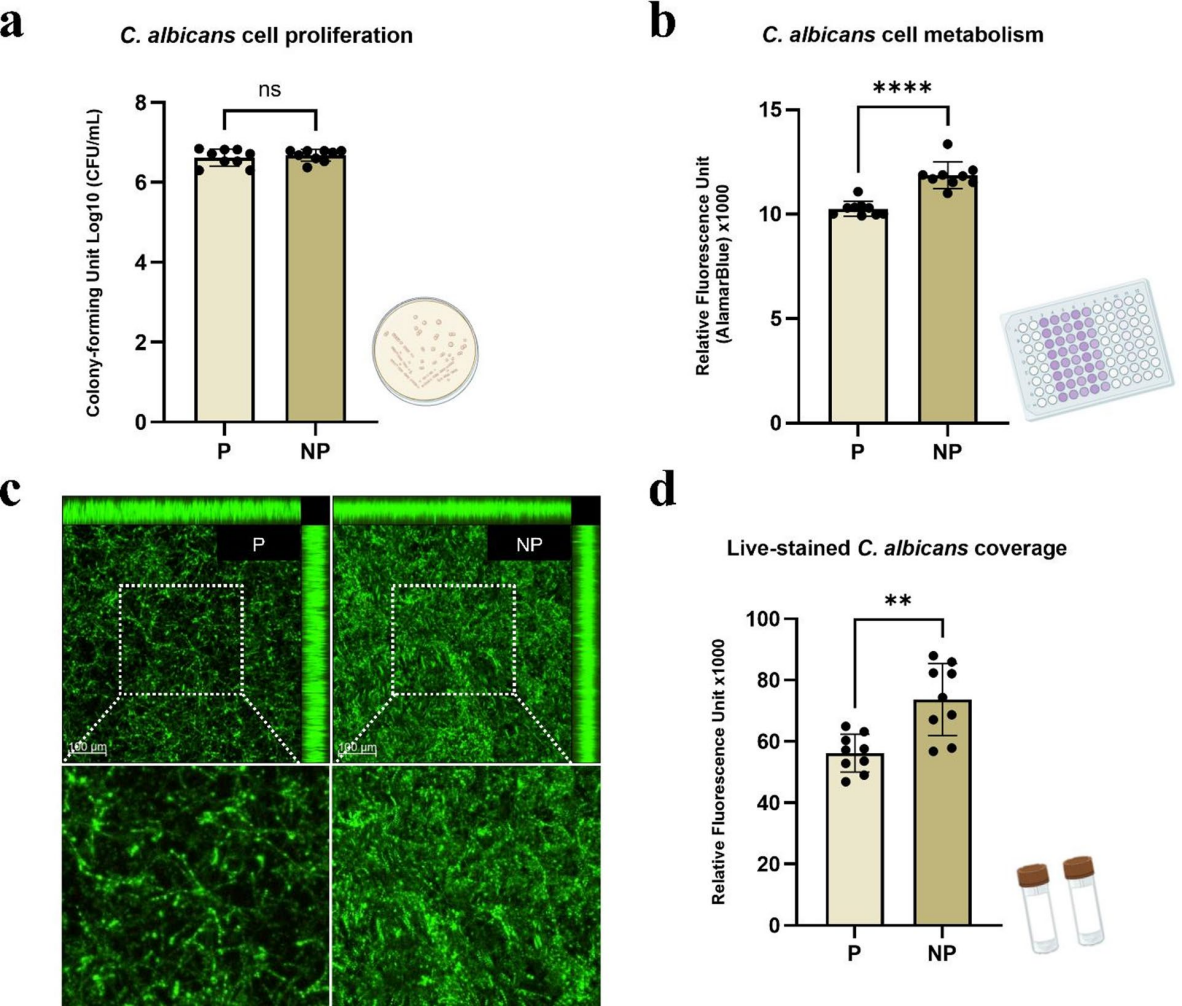
**a** Log10 CFU/mL values of *C. albicans* biofilm. **b** Metabolic activity assessed by AlamarBlue assay (relative fluorescence units, RFU). **c** Representative CLSM images of *C. albicans* biofilm coverage (live cells in green) at 10× magnification (scale bar: 100 μm). **d** Quantification of live biofilm coverage based on fluorescence intensity. *Note.* P: polished group; NP: not polished group. ****p < 0.001; **p = 0.001; ns: non-significant.

For the analysis of *C. albicans* proliferation, colony-forming units (CFU/mL) were quantified. Both groups showed growth of approximately 6 logs, with no statistically significant difference (*p* > 0.05) (Fig. 3a). Cell metabolism assessed by the AlamarBlue assay resulted in 10,262 ± 354 a.u. (P) and 11,870 ± 639 a.u. (NP), indicating a significant reduction in metabolic activity after polishing (*p* < 0.05) (Fig. 3b). For *C. albicans* biofilm coverage on the samples, quantitative and qualitative analyses were performed using three representative confocal images per group to obtain fluorescence intensity values. CLSM images revealed a denser green-stained biofilm on NP surfaces compared with P (Fig. 3c). Fluorescence intensity measured over a standardized area of 405,388.2 µm^2^ confirmed this statistically significant difference, with mean values of 56,200 ± 6,200 a.u. for P and 73,700 ± 11,800 a.u. for NP (*p* < 0.05) (Fig. 3d).

**Fig. 4:**
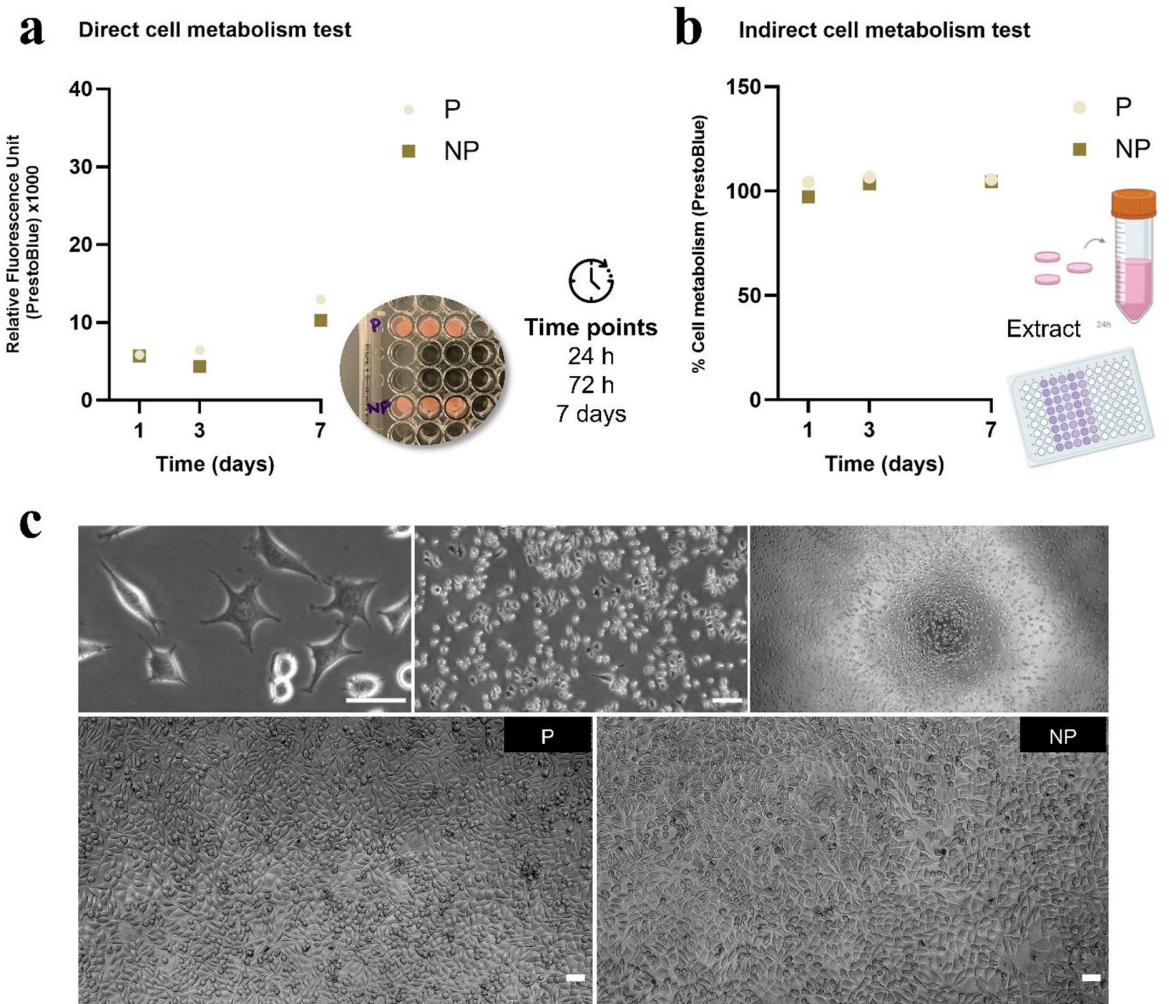
**a** Relative fluorescence units (RFU) from the direct cell metabolism test at 1, 3, and 7 days. **b** Percentage of cell metabolism from indirect cell metabolism test, normalized to the control group, both assessed by PrestoBlue assay. **c** Representative images of L-929 cell morphology and proliferation 24 h after seeding (top) and monolayer formation after 72 h of extract exposure in polished (P) and not polished (NP) groups (bottom) (scale bar: 50 μm). *Note.* P: polished group; NP: not polished group.

Cytotoxicity assessed by direct contact showed RFU values of 5,845 a.u. (P) and 5,727 a.u. (NP) on day 1, increasing on day 3 and reaching 12,982 a.u. (P) and 10,267 a.u. (NP) on day 7. Both groups exhibited approximately a two-fold increase in fluorescence from day 1 to day 7 (Fig. 4a). In the indirect contact test, results were expressed as percentage of cell metabolism normalized to the control group (cells incubated in culture medium). Metabolic activity in both experimental groups was comparable to the control, consistently around 100% over 7 days (Fig. 4b). In the qualitative analysis of the indirect contact test, representative images showed L-929 cells at a sub-confluent stage 24 h after seeding. After 72 h of extract exposure, cells reached high confluency with monolayer formation and displayed elongated fibroblast-like morphology (Fig. 4c).

**Fig. 5:**
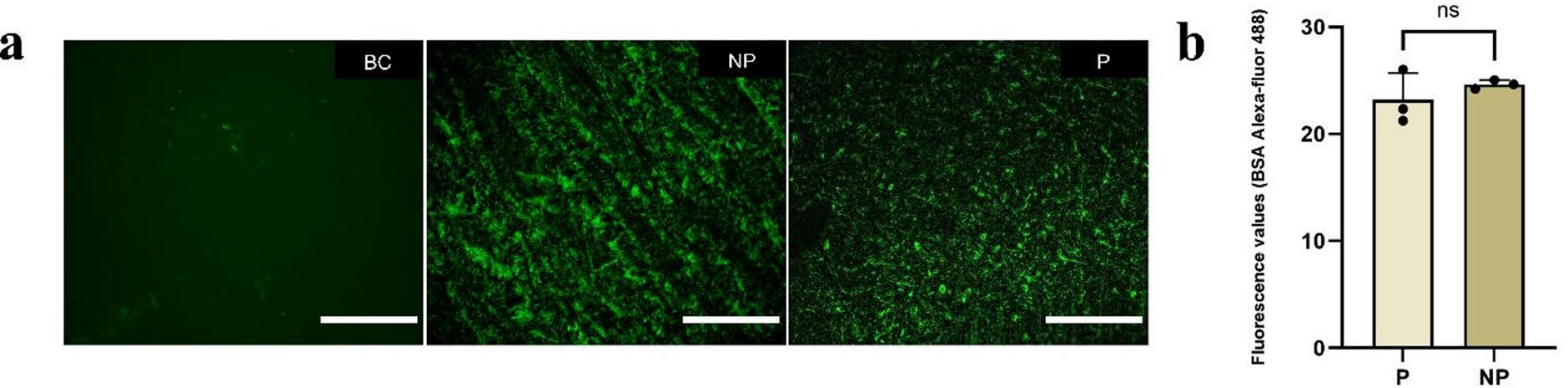
**a** Representative fluorescence microscopy images of Alexa Fluor 488-labeled BSA on the sample surfaces, acquired at 20× magnification (scale bar: 50 μm). **b** Quantification of fluorescence intensity based on representative images. *Note*. BC: background control; P: polished group; NP: not polished group. ns: non-significant.

Protein adsorption analysis showed a uniform distribution of BSA in the polished group, whereas the not polished group exhibited localized regions of higher accumulation (Fig. 5a). Autofluorescence background from the samples was checked and no signal was detected. Quantitatively, after background subtraction, fluorescence intensity measured over a standardized area of 133,519.6 μm² from three representative images per group resulted in mean values of 23.19 ± 2.50 a.u. (P) and 24.63 ± 0.40 a.u. (NP), with no statistically significant difference between groups (*p* > 0.05) (Fig. 5b).

## Discussion

This study evaluated the effects of surface polishing on an LCD-printed denture base polymer, focusing on surface roughness and its association with mechanical and biological responses. The null hypothesis was partially rejected, as polishing resulted in a significant reduction in roughness, while mechanical properties remained unaffected. In addition, polished surfaces demonstrated lower *Candida albicans* metabolic activity and live coverage, and cytotoxicity and protein adsorption were not significantly altered, suggesting this procedure did not adversely affect the biocompatibility of the material.

The surface roughness of denture base favors microbial adhesion, which under predisposing conditions such as systemic disease or poor hygiene may lead to denture stomatitis^1,12^. Because *Candida albicans* can act as an opportunistic pathogen, reducing the surface irregularities of prosthetic bases can represent an important preventive strategy. Various studies have emphasized the benefits of minimizing surface roughness through polishing protocols, as smoother polymers surfaces accumulate fewer microorganisms and facilitate routine cleaning^9,41,42^.

The roughness values of the not polished samples (NP) varied from previous reports comparing LCD-printed samples with conventional, DLP-printed and milled samples^43–47^. This suggests that surface properties are influenced by intrinsic material characteristics and processing factors such as printer technology, printing orientation, layer thickness, and post-processing procedures^31^. To ensure comparability, the present study employed a 90° printing orientation, which has been reported to reduce roughness and *Candida albicans* adhesion, representing the most favorable clinical condition, while also improving accuracy^31,48,49^.

In the context of 3D printing methods, LCD printing technology have been associated to disadvantages related to low resolution and accuracy^47,49^. However, the studies are controversial as it has been reported that DLP printers generally produce smoother surfaces, with Ra values around 1 µm^44^. Conversely, Viotto et al.^46 reported lower roughness in LCD samples than in DLP^, despite methodological differences, including a polishing standardization step prior to coffee immersion.

Both contact and non-contact profilometers yielded different absolute values but consistently demonstrated a significant reduction in surface roughness. The contact profilometer physically traces the surface profile, whereas the non-contact laser microscope captures the topography optically, allowing comparison with previous studies^9,29,35^. Importantly, the proposed polishing protocol achieved the clinically acceptable threshold associated with reduced microbial adhesion^25,50^, highlighting its practical applicability as a procedure that can be further replicated chairside or in the laboratory.

Improvements in polymers smoothness after polishing have been consistently documented^41,51,52^, and Kraemer Fernandez et al.^53^ showed that polished DLP‑printed samples had better surface characteristics than coated samples. Similar reductions in Ra have also been reported following mechanical polishing of 3D‑printed polymers^54,55^, although Quezada et al.^55^ found lower values with manual than mechanized polished samples. Nevertheless, discussion is still limited due to the lack of studies on polishing effects in LCD‑printed polymers, indicating the need for further investigation in this area.

Because denture base materials must resist localized compressive loads in areas of mucosal contact during mastication, compressive testing was performed to evaluate their strength and durability, critical properties for long-span prostheses^34^. Mechanical testing showed no significant differences between polished and not polished groups, consistent with previous findings on acrylic resin, where controlled polishing did not induce structural or chemical degradation^55^. The polishing procedure likely affected the superficial layer without compromising the integrity of the internal structure.

Rougher surfaces generally favor *Candida* colonization, as irregularities create retention niches that protect cells from shear forces during cleaning^15,42^.  Although this study did not test different materials, several studies have shown that 3D-printed polymers exhibit higher microbial adhesion than PMMA and milled CAD-CAM samples, along with increased expression of virulence factors such as hyphal transition^5,56,57^. Consistent with these observations, Ryu et al.^57^ demonstrated that high roughness and consequent microbial adhesion of this material, particularly across different printing orientations, require meticulous polishing and regular cleansing.

In this study, polished samples did not significantly reduce CFU counts but altered *C. albicans* metabolism and live coverage on the samples’ surface. Such discrepancies may be influenced by methodological differences, since CFU assays rely on colony counting, which have been suggested to potentially underestimate the number of cells per mL^58^. The observed reduction could also be related to the decreased contact surface after polishing, which may limit the number of adhered cells. Conversely, rougher surfaces provide a larger contact area that favors cell adhesion^13,59^.

Other surface properties, such as wettability and surface free energy, may also influence microbial adhesion^15,60^. In this study, polished samples showed reduction in *C. albicans* metabolism and slightly higher water contact angle values compared to not polished surfaces. These differences, however, were modest and limited and did not establish a clear link with previous reports^53,61^ that associated rough and hydrophobic surfaces with enhanced microbial adhesion, as well as with complementary literature suggesting that hydrophobic materials can promote *C. albicans* biofilm formation through non-covalent interactions between cell wall proteins and the substrate^17,42^.

According to the Wenzel model^18^, surface roughness can modify wettability depending on the intrinsic character of the material. In this study, it was suggested that polishing might reveal the hydrophobic nature of the polymer. However, this measurement alone cannot fully characterize hydrophobicity, as increased surface contact in rough samples may facilitate drop spreading. Moreover, the literature remains inconclusive regarding the association between roughness and wettability, as well as their impact on biological outcomes^9,62^.

Physicochemical factors, including surface charge and polymer composition, can influence protein adsorption and thereby affect cell and microorganism adhesion^63,64^. Early adsorption of salivary proteins is known to mediate the initial stages of biofilm formation^65,66^. Daeschel et al.^67^ reported that protein adsorption to solid surfaces can be modulated by temperature, pH, protein characteristics, and surface properties. In the results, no significant differences in protein adsorption were observed between groups. This may be related to nanoscale protein organization influencing its adsorption independently of surface topography, an aspect not assessed in this study and deserving further investigation.

Since the denture base is the primary interface with the oral environment and in prolonged contact with the mucosa, such modifications could affect its biocompatibility, particularly as 3D printing polymers are mainly composed of methacrylate or acrylate monomers, that present potential toxicity^68–70^. The methods applied demonstrated sustained cytocompatibility, with metabolic activity comparable to the control group (100% viable cells) over time, although the assays may not detect subtle physiological changes. In line, Dai et al.^71^ evaluated the polishing of DLP‑printed samples and observed that polished samples did not exhibit additional cytotoxicity.

In clinical routine, the internal surface of dentures is usually left unpolished under the assumption this procedure could reduce retention. However, this property depends primarily on border and posterior palatal seals rather than the microtexture of the support tissue^7^. Although retention was not directly assessed in the present study, Neeraja et al.^8^ reported that smoother surfaces yielded higher retention values, suggesting that increased roughness may disrupt adaptation and compromise adhesion and cohesive forces. Since the intaglio surface is the primary site of denture stomatitis, this procedure is relevant for future studies evaluating clinical outcomes related to 3D-printed complete dentures.

This study has some limitations, including the use of only one polymer and printing technology, the absence of a mechanical polishing device, the use of flat samples that do not reproduce the complex geometry of complete dentures, and reliance on a single-specie biofilm and a single cell line in the cytotoxicity test without assessment of inflammatory or oxidative markers. Further investigations incorporating multispecies biofilm models and additional biological evaluations would help to strengthen the evidence base and broaden the applicability of the proposed protocol.

## Conclusion

The results indicated that surface polishing reduced surface roughness and was associated with lower *Candida albicans* biofilm metabolism and coverage on LCD‑printed denture base samples, while mechanical properties and cytocompatibility were not significantly affected.

## Supplementary Information

Below is the link to the electronic supplementary material.

Supplementary Material 1

## Data Availability

Data are provided within the manuscript or supplementary information files.
