# Supplementary material for: Effect of surface polishing on roughness, biofilm formation, and biocompatibility of LCD-printed denture base polymer
Source: Sci Rep. 2026 Mar 31;16:15577. doi: 10.1038/s41598-026-45942-y (PMC13187404; doi:10.1038/s41598-026-45942-y)
Supplement: Supplementary file 1 — Supplementary Material 1 [file 41598_2026_45942_MOESM1_ESM.docx]

**Supplementary Information**

**Table 1.** Composition/information released by the company.

| **Resins** | **Color** | **Representation** | **Composition** | **Manufacturers’ post-processing instruction** |
| --- | --- | --- | --- | --- |
| Cosmos Denture Base  Yller Biomaterials, Brazil | Pink | CD | Oligomers, monomers, photoinitiators, stabilizer, pigment | Wash the objects in isopropyl alcohol or ethanol using two consecutive baths of 5 min each. Post-cure in a 72 W UV chamber for up to 10 min |


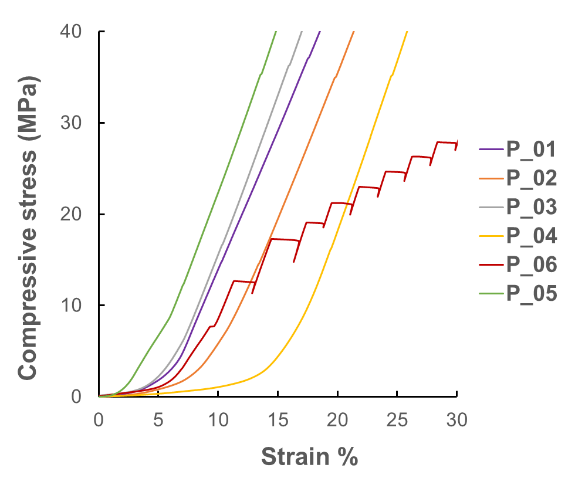

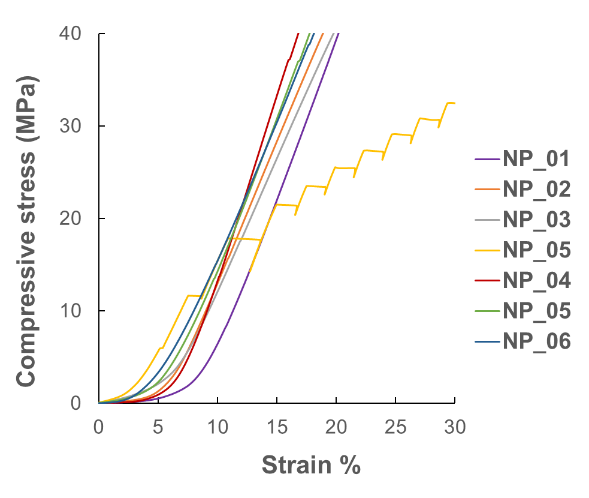


**Fig. 1**. Individual compressive stress–strain curves of P and NP specimens. Each curve represents one sample tested under compression. Note: Curves from specimens P_06 and NP_05 were excluded from the analysis due to anomalous measurement behavior.
